# Understanding Household Behavioral Risk Factors for Diarrheal Disease in Dar es Salaam: A Photovoice Community Assessment

**DOI:** 10.1155/2011/130467

**Published:** 2011-09-28

**Authors:** Natalie Badowski, Cynthia M. Castro, Maggie Montgomery, Amy J. Pickering, Simon Mamuya, Jennifer Davis

**Affiliations:** ^1^Stanford Prevention Research Center, Stanford University School of Medicine, 1070 Arastradero Road, Suite 100, Stanford, CA 94304-1334, USA; ^2^Civil & Environmental Engineering Department, Stanford University, Stanford, CA 94305-4020, USA; ^3^Emmett Interdisciplinary Program in Environment and Resources, Stanford University, Stanford, CA 94305-4020, USA; ^4^School of Public Health, Muhimbili University of Health and Allied Sciences, P.O. Box 65015, Dar es Salaam, Tanzania

## Abstract

Whereas Tanzania has seen considerable improvements in water and sanitation infrastructure over the past 20 years, the country still faces high rates of childhood morbidity from diarrheal diseases. This study utilized a qualitative, cross-sectional, modified Photovoice method to capture daily activities of Dar es Salaam mothers. A total of 127 photographs from 13 households were examined, and 13 interviews were conducted with household mothers. The photographs and interviews revealed insufficient hand washing procedures, unsafe disposal of wastewater, uncovered household drinking water containers, a lack of water treatment prior to consumption, and inappropriate toilets for use by small children. The interviews revealed that mothers were aware and knowledgeable of the risks of certain household practices and understood safer alternatives, yet were restricted by the perceived impracticality and financial constraints to make changes. The results draw attention to the real economic and behavioral challenges faced in reducing the spread of disease.

## 1. Introduction

In the past decade, with international support, Tanzania has made substantial efforts to expand water supply and sanitation infrastructure [1]. Despite the increasing coverage, the child mortality rate in 2009 (under age 5) was 108 for every 1000, and nearly 13% of those deaths were attributed to diarrheal diseases [2]. Even with improved, uncontaminated sources for drinking water, human behaviors contaminate the household drinking water supply and promote pathogen transmission. Studies from many developing countries showed that microbiological contamination increases significantly between the source point and the point of use in the household [3]. A recent study in Tanzania indicated that the clean drinking water obtained from community borewells became contaminated once it was stored in people's homes and was associated with greater levels of fecal contamination on family members' hands [4].

A number of methods have been used in Tanzania and other countries to explore sources of household level contamination. These studies have repeatedly described the deterioration of household drinking water [5], drinking water contamination [6], and increased microbial counts in drinking water [3]. However, these studies have been unable to associate household drinking water contamination levels with hygiene practices, water handling, or sanitation practices. Similarly, studies that used survey or oral report methods to ascertain the root of the problem have been unable to link behaviors to this subsequent contamination. As described by Boerma and Sommerfelt [7], the self-report approach is largely limited by social desirability and recall bias, as most people report the more socially desirable or less-stigmatized course of action, rather than their actual practices, and they may not readily recall all the components of their daily routine that lead to contamination. 

One way to gain perspective on household habits that may increase risk of disease transmission is to have family members document their own daily routines without intrusion from the researcher. This method may be less invasive from the household's perspective, and more likely to reduce respondent bias and socially desirable reporting. With the Photovoice method, the participant provides his or her point of view by taking photographs and providing narratives to describe what they see. It is an accessible method that bridges cultural barriers and engages the community to drive the information gathering. Wang et al. demonstrated the first codified use of Photovoice with 62 Chinese women who captured their everyday life, health, and work reality [8, 9]. Their stories and photographs revealed their first-person account of women's daily challenges and helped initiate a number of policy changes including day care centers and midwife training facilities. While Photovoice has traditionally been as a catalyst for policy changes, it is also a powerful qualitative research tool. Photovoice studies have assessed the housing concerns of homeless African American women [10]; the needs of post-hospital discharge elderly patients [11]; the barriers and facilitators to walking in the elderly [12]; the accessibility of healthy food to New York women [13]; the health concerns of young Latinas [14]. The method has also been used by Matheson et al. [15] for studying household dynamics and food preparation methods that contribute to obesity among Latino communities and to identify the perceived lack of infrastructure and financing difficulties perceived to be more important than the health threat of contaminated water in the management of the water filtration system in Limpopo, South Africa [16]. 

The purpose of the current study was to use modified Photovoice methods to understand household practices around water and sanitation that may contribute to household stored water contamination in peri-urban settlements of Dar es Salaam, Tanzania. The study aimed to discover current hygiene, water, and sanitation practices among household members, to understand family members' challenges and facilitators to implementing recommended practices, and to identify possibilities for future intervention.

## 2. Methods

The research design was a qualitative, cross-sectional study using modified Photovoice methodology to explore the daily activities of Dar es Salaam household members (particularly mothers) that may create greater risk for microbial water contamination and diarrheal disease. The project followed Photovoice methodology as outlined by Wang et al. [8, 9] and the Photovoice manual [17], adapted for more recent research standards [18], and incorporated individual interviews and a larger number of picture narratives. 

### 2.1. Participants

The sample consisted of 13 households recruited from 2 communities in peri-urban areas of Dar es Salaam. Specific communities were selected because they have comparable socioeconomic and demographic characteristics and equivalent water supply and sanitation services (e.g., access to government-supplied deep borewells and private or shared latrines [4]). The peri-urban areas were selected due to the recent installment of municipal community water sources (such as the borewells from the Community Water Supply and Sanitation Program initiative). When the study was conducted in 2008, 80% of urban, 45% of rural, and 54% of the total Tanzanian population were served by these “improved” water sources, thus, the peri-urban areas reasonably represented typical water access [19]. The criteria for a participating household were a female head-of-household between the ages of 18–50 with at least one child under the age of 5 and the presence of an older child or other household member willing and able to photograph the mother. In exchange for participation, households were given an album of their pictures and a kilogram each of tea and sugar (equivalent to $1.80 USD). Approval for the use of human participants in research was obtained from Institutional Review Boards of Stanford University and Muhimbili University of Health and Allied Sciences and the Tanzania Commission for Science and Technology.

### 2.2. Procedures

The researchers were formally introduced to the executive ward officers in each community through a liaison with a local nongovernmental organization. The ward officers then facilitated the identification of households eligible for the study. Households were recruited via face-to-face visits led by the ward executive officer or health ward officers and mediated by Muhimbili University of Health and Allied Sciences (MUHAS) students serving as research assistants and translators. These households were given information about the project and interviewed for eligibility and interest in participating. Oral consent was obtained from the female head of the household and either the male head or an adult family member (the parents had to provide consent for a child's participation), with members being asked for consent to use the photographs for research purposes and record their interviews into electronic audio files. Of the 15 households approached, two households refused to participate because of the recorded nature of the interviews. Of the remaining 13 households that provided consent, the photographers were the oldest daughter (5 households), a grandmother (1 household), an aunt (1 household), the husband (4 households), or a neighbor mother (2 households, in which the neighboring women photographed each other). These participants were then oriented to the task of photographing the daily activities of the mother of each respective household. 

Households were given a digital Kodak EasyShare camera (Eastman Kodak Company, NY) and trained in the basics of photography. The participants were given verbal instructions and hands-on demonstrations in the basics of camera use (e.g., turning the camera on and off, framing, and taking a picture) and the practical aspects of photography (e.g., keeping fingers away from the viewfinder, holding the camera steady). They were given minimal advice in order to minimize inhibition of their creativity and expression. The participants were trained until they demonstrated the ability to use the camera and were given brief, illustrated instruction sheets on camera use for reference. Next, the participants were told to focus their photography around “the essential events and routines of the mother's day.” They were broadly encouraged to follow the everyday activities of the mother. In order to minimize self-consciousness and bias, the participants were encouraged to just focus on photographing their usual routine and were not given instructions to specifically photograph any particular activities or events.

Participants were given 24 hours to photograph the mother's daily activities. After the 24 hours, the cameras were collected, the pictures were downloaded, and 8–12 photographs from each household that were indicative of daily routines were printed. The research team developed specific questions for each picture to explore the topics of water use, sanitation, hand hygiene, and health in the household. Additional photos (family portraits, pictures of the home, and surrounding property) were also printed and given to families in an album as a token of appreciation for participating in the project. 

Two days after photographs were taken, the research team returned to the participants' homes with the printed photographs and interviewed only the photographed mother in each household about the photographs (regardless of who took the picture). In these sessions, the mothers were asked to look at the pictures and describe the scene and associated thoughts, feelings, and issues connected with each picture. The participants were encouraged to tell stories about their pictures and to discuss the meaning of their photographed activity within their households. With the participants' permission, all of the responses were recorded with a digital recorder for subsequent transcription, translation, analysis, and reference. The interviews were transcribed in Kiswahili, then translated into English, and cross-checked by a second translator.

### 2.3. Data Analysis

Both qualitative and quantitative information was derived from the participants' photographs and their responses to interview questions. The participants took a wide range of photographs (from 9 to 56); 8–12 of the most “representative” photographs of daily routines were chosen for each household. These photographs were chosen by removing portraits, family photos, and redundant photos of the same subject matter. If multiple pictures of the same activity were taken, only one of the series was chosen to encourage a discussion on a variety of topics. In each case, 8–12 photographs remained that were used to create the questions and to direct the interviews. To derive quantitative data, the photographs were analyzed by the researchers and their main themes were identified independent of the interview content (i.e., the objects, people, and activities that were most commonly photographed). These themes were categorized and subcategorized based on recurrence and prevalence. All of the themes that emerged were described, quantified, and illustrated with exemplary pictures. 

The interview transcripts were processed using NVivo qualitative software (Version 8, QSR International, Cambridge, Mass) to organize, codify, and analyze the text data from the interviews. The 13 transcripts were examined and coded for themes. A number of main themes emerged from the narratives, chosen based on the words and phrases that appeared most often as analyzed with the NVivo software, which were further categorized into subgroups based on recurring ideas. All of the themes were likewise described, quantified, and illustrated with direct quotes.

## 3. Results

**Figure 1:**
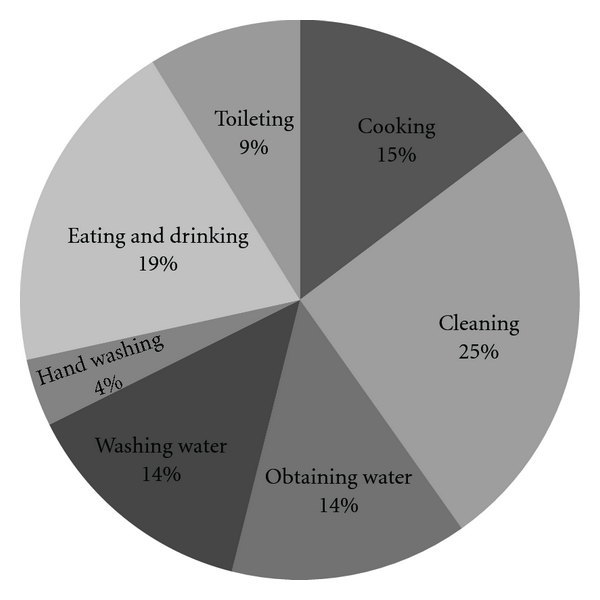
Distribution of common images in photographs (total photographs = 127).

A total of 127 photographs from 13 households (8–12 pictures from each household, yielding an average of 9.8 pictures per household), and 247 minutes of total interview time were analyzed. See Figure 1 for a distribution chart of common images that were documented based solely on visual inspection of the photographs. 

### 3.1. Toilets

Three of the 13 families took pictures of their household toileting facilities. Two were pit latrines, and one had a toilet stool connected to a bowl. Nine of the households discussed toilets in their interviews, making 31 direct references, and many more in conjunction with problems and challenges in their communities. In addition, there were 4 pictures and 6 references to child training potties from 4 households, with 2 of the pictures and references illustrating the use of diapers. 

A common theme in the toilet pictures and interviews was the self-described lack of a “proper latrine.” Family 1 discussed their facilities saying “It shows the type of bathroom we have and I know that it is not up to standard, but because I do not have the money to build a standard bathroom, we end up using such a bathroom.” When asked what a standard bathroom looks like, the mother commented “As you know, nowadays there are bathrooms having cement floors, and they have a toilet, they have a sink, and also they will have a tap…I think that if I have money, I will be able to improve it.” Likewise with the toilet, the mother commented “It is not a good toilet. It does not have a covering and the surroundings are not that good, but it is all because I do not have money.” 

**Figure 2:**
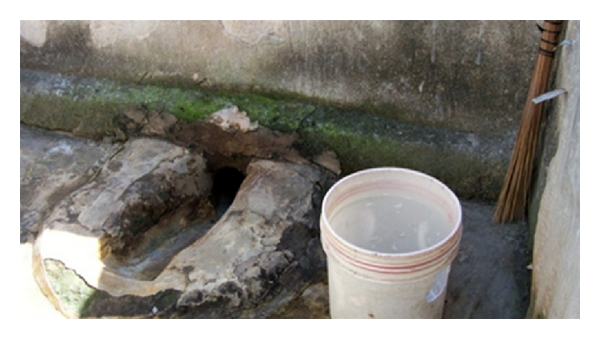
Example of a typical toilet. The water is used for cleaning the latrine, rather than for washing hands.

Several of the families mentioned variable use of water with toileting. In household 4, the photographer elaborated “When somebody goes to the toilet to help themselves, they need to take a lot of water with them but others take a small amount of water with them and they leave the feces unflushed and when a kid comes to defecate, they also leave their feces, so then the place becomes surrounded with dirt.” Of the three households that took pictures of toilets, only one picture captured water in the area and none captured soap. However, the water located near the toilet is not necessarily used for flushing or hand washing. When household 3 was asked “What is the bucket and the broom used for?” the family replied “For cleaning the latrine.” (See Figure 2.) 

#### 3.1.1. Child Toileting

**Figure 3:**
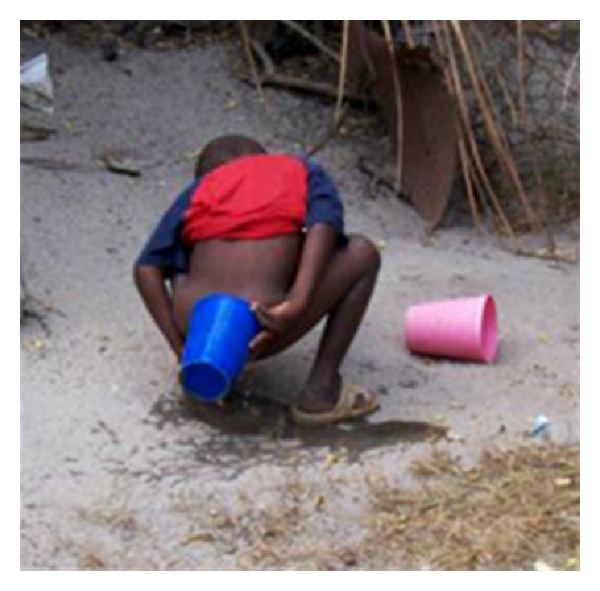
Picture of a child defecating outside. This is a common practice due to fears of children falling into the toilet holes.

Children's use of toilets and latrines was the focus of a considerable amount of discussion. One prominent complaint was that the hole of the toilet or latrine is too big for children's use. As a result, the children in some families defecated and cleaned themselves in the open, as demonstrated in Figure 3 below. When Family 1 was asked “Why did he [the child] defecate outside and not at the washroom?” the mother responded “Because they are small. The hole we use for the toilet is too big, so the kid might fall in.” When asked “Do you think that it might be a problem to health if they defecate outside?” the mother responded “I do not think it is so much of a risk because we collect the feces and we deposit them into the toilet.” 

In other households, a small plastic “potty” was the preferred means of child toileting. According to interviews, the potties are invariably cleaned with soap and water, and the feces are disposed of in the adult toilet. Effort is made to clean these potties thoroughly, with household 4 elaborating “I washed her with water and soap. After that, I throw the dirty water in the toilet and then I wash my fingers with soap and water. Then I dress my child and allow her to play.” 

**Figure 4:**
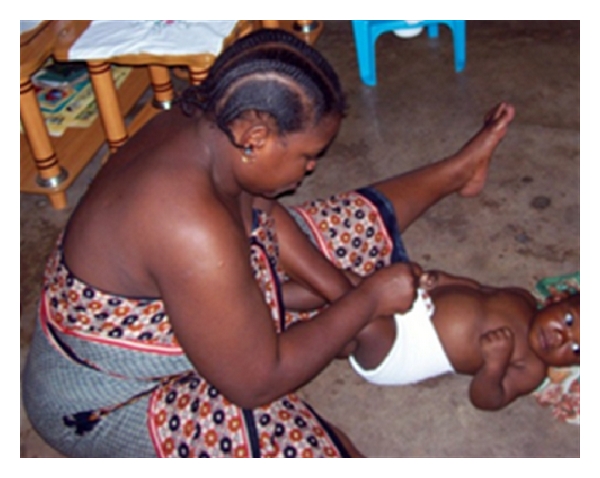
Mother changing her child on the floor for safety concerns. However, this same floor can be used for meal preparation and consumption.

The last child toileting practice observed was the use of diapers and diaper changes. In at least one household (household 10, see Figure 4), children have their diapers changed on the floor. When questioned about the practice, the mother explains that “I dress the child (change the diaper) on the floor because when you dress on the bed the child tends to jump up and fall down. So, I decided to sit on the floor and dress the child for safety.”

### 3.2. Hand Washing

**Figure 5:**
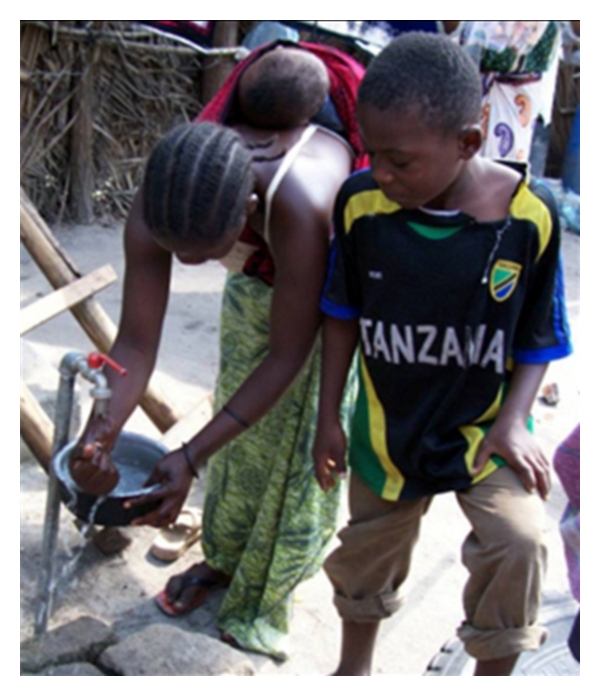
Example of handwashing after cooking. “Handwashing” is commonly a practice of having hands placed under running water.

**Figure 6:**
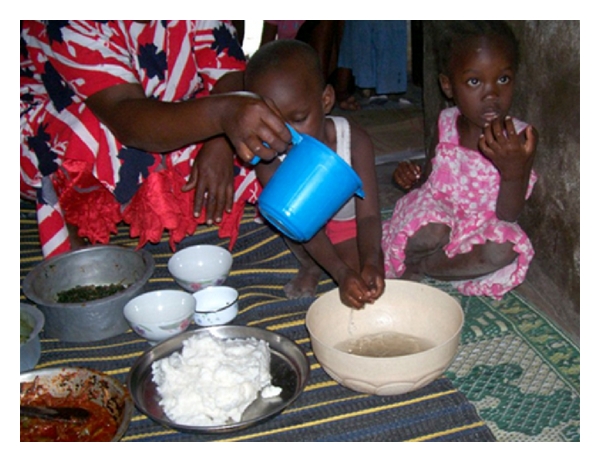
Washing hands before mealtimes. Note the absence of soap or soapy water.

Four families photographed themselves or family members washing hands. In the interviews, 9 different families made a total of 30 references to hand washing behavior. Households 1 (see Figure 5), 2, and 12 (see Figure 6) documented and photographed hand washing practices, hoping to demonstrate their hygiene practices, with household 12 emphasizing: “Even if they take fruits, tea, in everything I wash their hands…I insist for them to wash their hands so that they will not eat while their hands are dirty.”

Families mention hand washing activity before and after meals (households 1, 2, 6, 7, 9, 11, and 12), before feeding children (household 10 and 11), before meal preparation (household 11), and confirm hand washing behavior when asked directly (household 10). In the interviews, families did not mention hand washing after toileting or before or after any other activity (farming, animal handling, play, etc.). The other notable detail is that all the pictures demonstrate “hand washing” as a practice of either pouring water over hands or that of dipping hands into a communal bowl. Soap was not observed in any photographs of hand washing.

### 3.3. Water

**Figure 7:**
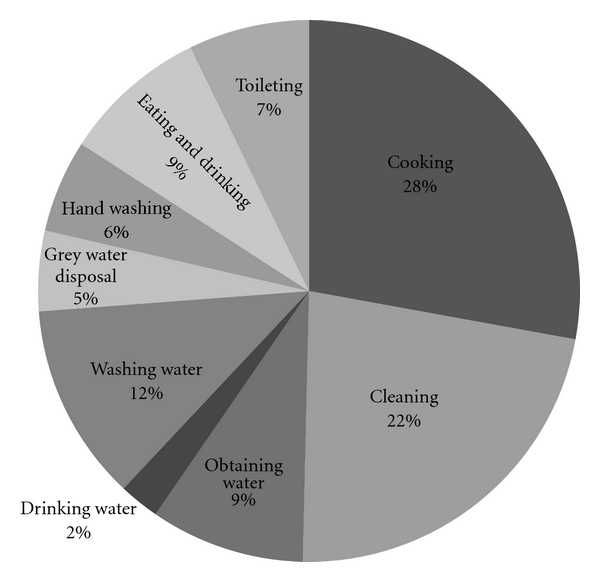
Distribution of the number of verbal references to particular topics in the photovoice interviews.

There are pictures and references to water from all 13 households. Water is seen throughout many household activities. As seen in Figure 7, we found pictures and verbal references to cooking with water (with 15 photos from 9 households and a total of 151 verbal references from all 13 households); general cleaning (5 pictures from 4 households and 41 verbal references from 12 households); washing dishes (14 pictures from 9 households and 64 verbal references from all 13 households); the task of obtaining water (14 pictures from 8 household with 50 verbal references from 12 households). As household 5 points out, “water is used for all the activities.”

#### 3.3.1. Obtaining Water

One common theme in the interviews centered around obtaining water and the different water sources used. Each of the 14 pictures from the 8 households pictured the mother filling some sort of container, either with water from a well or a tap. Of the 13 households, 6 used a public tap, 6 used a private tap, and only one obtained water from a private well. All of the households had to pay for their water, with costs ranging from 20–50 Tsh (Tanzanian shillings) per 20 liters (0.01–0.03 USD) or 7,000–50,000 Tsh per month (4.38–31.29 USD). The mothers reported walking up to10 minutes to collect water, waiting in line with other households as long as 30 minutes to obtain water at its source, and making 2–5 trips for water per day.

#### 3.3.2. Separating Water for Different Purposes

Another common theme in the interviews was the effort to treat “types” of water differently. Many households categorized their water based on its use: cooking water, bathing water, water for animals, children's water, water for gardening (at least one reference per household), and drinking water (the largest category with 13 references from 7 households). The households also designate specific water sources for particular household tasks, using municipal tap water (63 references from 13 households) for drinking water, and personal well water for washing activities (households 1 and 7). The households also designate specific utensils for particular uses, pointing to a container used only “for keeping water for the chickens” (household 6) or a bucket “only used for fetching water for bathing” (household 5).

#### 3.3.3. Drinking Water

Some families documented keeping drinking water separate from stored water used for other purposes. Household 3 demonstrated the storage of water in a modern refrigerator with a special cup designated to pour water for drinking. When asked if there is any chance of the water becoming contaminated, the family explains “I do not think so, because the bucket is normally inside the refrigerator and there is a cup tied with a short string to the bucket. In case someone wants to get water from it, someone will use that cup, but they will not be able to drink from the same cup, because the string is very short. So they need to put it into a separate glass or cup so that they can drink it.” The same family also stores boiled water separately for children and designates a specific cup for children. 

Drinking water is also generally the only water that is potentially treated. Two of the households (10 and 12) reported filtering their drinking water though the filtration method was not reported. Households 8 and 9 add chemicals or “medicine” to their water but could not name the substance used. Household 7 boils the water used for drinking. Two households use a combination of techniques to keep the water safe (chemicals and boiling in household 9; filtering, chemicals, and boiling children's water in household 3). 

In contrast, 7 of the households do not treat their drinking water in any way (households 1, 2, 4, 5, 6, 11, and 16), and only mention water boiling in the process of cooking and food preparation. There are a number of reasons for the lack of water treatment. Households 1, 2, and 3 state that they trust the water from the neighborhood tap, so they do not boil it. Household 3 also commented on the dislike of water treatment method, while household 5 pointed out the heat as a barrier to water boiling. At least one household commented on the expense of burning charcoal or wood for the preparation of boiled drinking water. Only one of the families expressly mentioned that their stored drinking water was not safe. Household 4 commented “…our door [to the house] is constantly open and the when the wind comes it comes with a lot of bacteria and dust, and get into water. And for us, we do not boil drinking water and its only God's mercy that we do not get sick.”

#### 3.3.4. Water Covering

**Figure 8:**
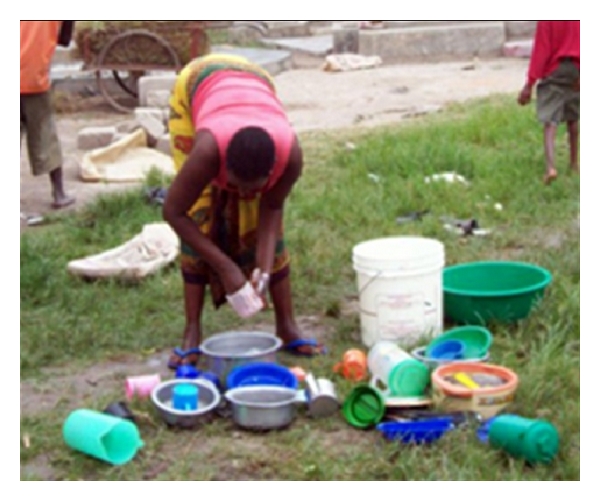
The common practice of leaving dishes on the ground to dry.

Another important theme that emerged from the pictures was water covering (or lack thereof). Though every family claimed storage of drinking water in covered containers, only 17 pictures showed containers with a lid and 9 showed uncovered containers with standing water. Three of the households (household 3, 4, and 5 photographed in Figure 8) stored drinking water in uncovered containers. In the interviews, 6 households directly mentioned water covering, one emphasized the importance of covering containers (household 7), and another mentioned the importance of covering shallow wells (household 8). Conversely, when probed for more information, a couple of the households discussed uncovered containers. Household 3 revealed “Because the containers are not covered, bacteria can contaminate the water, and when you come and fetch the water for any other activity, you can get a disease, such as skin diseases.”

#### 3.3.5. Water for Washing

Some families used unimproved and untreated water sources for washing. While most households use tap water for all activities, including dish washing, households 1 and 7 obtain water from nearby, unattended shallow wells. Family 1 discussed that “The [shallow] well was constructed for personal use as a toilet pit, but since it was not covered, we use the water for other means…. The water is not safe, so we do not use it for drinking or cooking, but we use it for other activities like washing.”

#### 3.3.6. Water Disposal

Another major theme that emerged from the interviews was that of the grey water disposal. Ten households (with 26 references) talked about “throwing out” or “pouring” out refuse water after any activity. Three pour it into a canal (after direct questioning, one admits that the canal flows into a neighboring household and another points out that it drains to the streets behind the house). The rest pour it out to a nearby area, for example, “under those fruit trees” (household 12) or “outside the house onto the dust” (household 16). All of these households dispose of water in this way regardless of activity (i.e., laundry washing and dish washing). In some cases, water is reused for other tasks. For example, Family 1 photographed their child bathing in a basin commenting “It is a picture of my grandchild taking a bath in a basin. We are washing the kid with the water we had used for washing, and then later we rinsed him with clean water.”

### 3.4. Food Disposal and Dish Washing

The analyses revealed 14 pictures of washing dishes (9 households) with 64 references from all 13 households. In every picture of dish washing where the water is visible, one can see soapy suds, indicating the use of soap. There was also 1 picture of active drying and 7 pictures of the air drying of dishes or utensils (6 households) with 17 references from 7 sources. The households vary in the method and timing of food disposal and washing. Leftovers are given “to the dogs and cat we have here” (household 7) or stored in “a special place” (household 9) or in a modern refrigerator (households 10 and 12). Household 11 mentioned throwing them away, stating “we put it in the bin. There are people who collect the trash.” 

Only one household (household 6) photographed immediate drying and putting away of washed dishes, while the others mentioned it (households 9 and 11), or used alternate methods. For example, households fill large basins with cleaned dishes (household 7) or let them dry on the ground in the sun (household 4, 5 (see Figure 8), and 10). 

Putting dishes on the ground to dry is done largely for convenience or the lack of alternative places to store dishes. As household 7 points out “I do not have a cupboard, so I store my dishes into those bins.”

### 3.5. Cooking and Eating

The most common theme in both the pictures and interviews was food preparation and cooking. Out of the 127 pictures, we found 4 of sorting/food preparation (3 households) with 11 references from 4 households, as well as 15 pictures of cooking (9 households) with 151 references from all 13 households. Most of the households discussed the process of preparing the traditional dishes such as ugali (boiled maize meal), mchicha (greens), and, occasionally, meat. Mealtimes were also documented for many of the families. There were 10 photographs from 7 different families centered on eating, feeding, and drinking. Similarly, there were 25 references from 8 households for eating/feeding and 22 references from 8 households about drinking.

A common practice seen in the pictures is that every family (except household 9) performs all the cooking and food preparation on the ground. The exception is the sorting of rice, beans, and greens, which was photographed performed seated in a chair. Every photographed meal was also taken on the ground; an area was cleared, a mat was set down, and all individuals sit down to a communal meal. Half of the pictures show this type of setup outdoors, while the other half show meals taken on the ground inside the house structure.

## 4. Discussion

The spread of diarrheal disease often begins with fecal contamination of water, hands, and food. Analyzing the photographs and interviews of 13 families revealed a number of avenues for potential contamination in the households. The pictures and interviews show that many of the households regularly wash hands and understand its importance for preventing the spread of germs. However, hand washing often only happens before meals and mainly consists of pouring water over the hands with inconsistent use of soap. While 10 households said that they use soap and water in hand washing and other activities, only 2 pictures show a physical bar of soap, and the only examples of soapy water were seen with laundry or dishwashing activities. Therefore, hand washing may not consist of a thorough scrub with soap and clean water that effectively removes pathogens. Likewise, there is a dearth of both photographed and self-described hand washing after toileting. While people verbalized the importance of hand washing with soap and water, this awareness does not translate to practicing recommended hand washing habits. This finding has been replicated in numerous studies, with hand washing with soap reported in only 24% of possible hand washing opportunities in Kenya [20] and in 3.5% of Ghanaian mothers after defecation [21, 22].

Our analysis also showed that many families were conscious of the importance of keeping their drinking water safe. However, the interviews reveal a number of discrepancies between knowledge and practice that may perpetuate health problems. There was a theme of assumed safety of water from municipal borewells and lack of acknowledgement of the likelihood that family members may recontaminate the water once it is in their possession. Water was handled after touching objects outside and after toileting, but hand washing did not always occur prior to handling the water; these gaps in hand washing consistency can easily contribute to water contamination. Another concern was the large number of photographs displaying uncovered drinking water containers despite most families' claims that they store water in a container with a lid. The reasons for uncovering containers were numerous, including allowing boiled water to cool or permitting easier access to drinking water. In these situations, practical considerations took precedence over health implications and awareness of alternatives for safeguarding drinking water. 

Whereas water safety and hand washing received much attention and discussion, household sanitation issues were overlooked by the majority of households as few photographed their facilities or voluntarily discussed management of human waste as an important daily activity. Among the few households that drew attention to their sanitation facilities, they were again quite knowledgeable about threats that poor sanitation poses to physical well-being. These households had a conceptualization of a “proper toilet” and expressed desire to have it yet were not taking steps to improve their sanitation infrastructure in the home. As the interviews show, safety concerns for children falling into pits have led them to openly defecate on the family property. In other families, these children use potties in common areas in close proximity to the home. Both of these practices introduce fecal material on the ground where the children later play. This is especially a concern when food preparation and consumption is performed close to the ground, and children's hands and household dishes frequently come into contact with the ground. Similarly, at least one household (household 10) demonstrated that children are changed on the floor. While the lack of a changing table necessitates the activity for safety reasons, this is the same floor that is often used for other activities such as play or food preparation. 

Another relevant issue was the disposal of grey water close to the home. In almost every family, water used for washing, whether clothes or potties, was disposed directly to the ground and poorly draining open canals. This practice is especially risky when so many activities (children's play, food preparation, cooking, and eating) take place close to or on the ground. 

### 4.1. Limitations

While PhotoVoice has the ability to probe into the inner workings of a household, the methodology is still limited in scope. The families photographed water sources and activities of daily life but might have tried to gear their pictures and interviews towards what they believed the researchers would want to see (my child washing his/her hands before mealtimes) rather than their actual practices, creating a bit of a “Hawthorne effect.” On the other side, certain behaviors may be so deep rooted in habit that it may be difficult to tease out specific motivations. As Scott et al. [22] pointed out “people may give reasons for the behaviours they are exhibiting, when interrogated, answers may represent the post-rationalization of behavior rather than true behavioural motivations.” In addition, people may have a specific motivation but do not express their reasoning due to social norms and taboos (especially around toileting behavior).

This study also includes only a cross-section of the population. We executed the Photovoice project with 13 families from two different communities. While we believe that this is a good cross-sectional sampling of these communities, there is always additional information that can be gathered from the inclusion of more families and communities. The methodology is also qualitative, which provides rich material for analysis, but which can be biased via participant responses and researcher analyses. Here, we provided the families with minimal instruction about the project to assess the spontaneous occurrence of water and sanitation-related themes, and we used a systematic and objective analysis of the pictures and interviews.

### 4.2. Implications

Despite the persistence of risky habits, there was a very high level of awareness of recommended practices for safe water storage, hand washing, and management of human feces; most families could identify and explain what could make them sick. Yet the knowledge was not consistent with actual behaviors. As many households discussed, the persistence of many of the risky habits stem from lack of practicality and finances to invest in better water and sanitation at the household level. Several families spend a considerable fraction of their income on water usage, particularly during the dry season. There is also the hidden cost of the time used to collect water and the necessity of making several trips to the water source. To make the process more efficient, families store as much water as possible, creating more opportunities for contamination during storage. In addition, families reported not treating their water because of inconvenience, expense, or the dislike of chemical taste. Others knew their latrines were poor but described costs as the limiting factor in improving their sanitation. At least 7 households (families 1, 2, 3, 10, 11, 12, and 16) mentioned costs as an important factor in their decision-making areas about household spending. For example, household 3 mentions that “It is expensive to buy charcoal or wood, so we do not have enough to use for boiling water every day,” while household 16 referred to the concerns of paying for food and school expenses at the same time as the more pressing spending decisions they have to make. In addition, the financial decision-making is most frequently controlled by the father or the male head of the household, even though the mother has to manage the daily resource demands of the household. 

In at least two comprehensive reviews, hand washing has been correlated with a reduction of diarrheal disease on the order of 42–48% [23] and 39% in high-income countries or 32% in low-income countries [24]. Clearly, this shows that hand washing behaviors do work in disease prevention. Yet, despite this knowledge, our study shows that health-based education and awareness-building are not sufficient catalysts for changing household behavior, as the families interviewed possessed the knowledge but knowingly engaged in less safe habits. Unfortunately, this is a theme that is replicated in many different studies. For example, after an extensive educational intervention in rural India, complete with Glo-germ demonstrations and rallies, the authors found no increase in hand washing activity at key times [25]. Similarly, Scott et al. [21, 22] found that health education was an ineffective route to behavior change. Instead, the authors found that the strongest motivators for hand washing with soap were nurturance (or the desire to care and protect children), social acceptance (including the potential to rise in society), and disgust of feces, latrines, and putrid smells (and to keep a neat and clean environment). In addition, the authors pointed out that disgust sensitivity was the factor most correlated with soap use. 

Similarly, nonhealth-focused motivators were extracted from the Tanzanian families in this study, and these motivators can be fruitful targets to create more lasting change. Making home upgrades more affordable and/or aligning recommended practices with nonhealth-related values may be more viable for motivating families to make changes. For example, modern fixtures suggest improved social status and upward mobility, and well-kept latrines prevent pests and odors and are less visually offensive. These more desirable social and sensory benefits were more pressing and motivating to families than reducing risks from invisible pathogens in the environment. 

One approach that has had some success in many countries is the implementation of community led total sanitation (CLTS) activities. In this approach, facilitators trigger a “collective sense of disgust and shame in community members” as they reveal and confront the practice of mass defecation and its negative health impacts on an entire community. [26] With calculations of the production of fecal material in a day, demonstrations of the ease of fecal contamination of food and water sources, and the emphasis of an unpleasant environment, this approach has ignited communities to take on measures to change social norms around fecal management, build better latrines, and promote better hand washing practices. While these methods have been primarily used to end open defecation, adaptations could be utilized to focus on latrine and hand washing station improvements.

Families expressed interest in making improvements if they were more financially feasible, given the limits of households' discretionary spending. Large-scale sanitation initiatives already appear to be gradually improving sanitation infrastructure and changing social norms in low-income countries [26, 27]. Perhaps more ground-level microfinancing opportunities (i.e., installed payments, credit opportunities, rebates, or other economic incentives) and greater supply of affordable improvements (modern fixtures, durable latrine materials) would motivate families to make more technologically advanced changes within their household. The CLTS approach calls upon recruitment of local craftsmen and tradesmen to promote development and production of sanitary hardware products, promoting an increase in jobs industry in a local area. In turn, marketing efforts promote their products and create consumer demand for the supply [26].

If lower cost options were introduced and coupled or marketed with the potential social/sensory benefits, perhaps uptake of improvements would increase. As Panter-Brick [28] astutely pointed out the success of an intervention needs to be culturally compelling, must engage local communities, and must appropriately fit within the social and ecological landscape to be successful. With further research, community engagement, and culturally sensitive and inquisitive work, behavior modification on a large scale is possible, with the potential to create lasting change in social and community practices.

## 5. Conclusions

Overall, the findings from this study yielded vivid information about the challenges to prevent the spread of fecal pathogens within the household. The potential routes of contamination are many and intermingled. The Photovoice interviews identified a number of behavioral practices that may perpetuate the transmission of pathogens through fecal contamination of hands and drinking water, including the lack of adequate toileting facilities (especially for children); inadequate hand washing method; drinking water recontamination through contact with hands; uncovered storage containers; inconsistent chemical and/or filtration treatment of drinking water; grey water disposal into close proximity to homes; hand and dish contact with ground likely to contain traces of fecal material.
